# Optimization of biocatalytic production of sodium gluconate using a dual-enzyme system

**DOI:** 10.3389/fbioe.2025.1607782

**Published:** 2025-08-18

**Authors:** Jialei Ren, Piwu Li, Xiaofeng Wei, Jianbin Wang, Chuanzhuang Guo, Keyi Liu, Junqing Wang, Xia Li

**Affiliations:** ^1^State Key Laboratory of Green Papermaking and Resource Recycling, Shandong Academy of Science, Qilu University of Technology, Jinan, China; ^2^School of Bioengineering, Shandong Academy of Science, Qilu University of Technology, Jinan, China; ^3^ Dongxiao Bioengineering (Shandong) Co., Ltd., Jinan, China

**Keywords:** dual enzyme method, sodium gluconate, technology optimization, response surface, dissolved oxygen

## Abstract

Sodium gluconate has a wide range of applications, including in the fields of construction, textiles, medicine, the chemical industry, and food, so the industrialized production of sodium gluconate is particularly important. However, the preparation process of sodium gluconate is not mature enough, and the production cost is high, which restricts the development of the industry. In this study, the optimization of process conditions for the catalytic production of sodium gluconate from glucose via a dual-enzyme system of glucose oxidase (GOD) and catalase (CAT) was investigated in detail. Factors such as pH, temperature, metal ions, enzyme addition, stirring speed, and aeration were examined. After optimizing these parameters through one-way experiments, the Box-Behnken design (BBD) was employed to refine the process further, focusing on stirring speed, enzyme addition, and aeration. The optimal reaction conditions were identified as follows: a reaction pH of 5.9, a reaction temperature of 38°C, enzyme addition of 0.2%, batch addition, 80% GOD at 0 h, 20% GOD at 2 h, stirring speed of 700 rpm, aeration amount of 1.2 vvm, and a tank pressure of 0.04 Pa. Under these conditions, the reaction cycle for sodium gluconate production was reduced to 7.75 ± 0.5 h. These optimized conditions significantly improve existing methods, offering a more efficient and cost-effective approach to sodium gluconate production. The findings provide valuable insights for scaling up biocatalytic processes, with the potential for a substantial industrial impact, particularly in reducing production costs and improving sustainability in the chemical and food industries.

## 1 Introduction

Sodium gluconate, an important polyhydroxy organic acid salt, finds wide application in construction, textile, medicine, chemical processing, and food industries due to its unique advantages. These include stable physical properties, low toxicity, and excellent biocompatibility, making it no obvious irritation to human skin and mucous membranes (Ramachandran et al., 2006). Besides, because of a number of hydroxyl groups in its chemical structure, contains, sodium gluconate has excellent water solubility and chelating properties (Han et al., 2018b). Moreover, because the sodium salt of gluconic acid is nonvolatile and non-dampers during storage, it can be stably preserved for a long time under dry conditions at room temperature. Gluconic acid, a naturally occurring organic acid, is widely distributed in living organisms such as plants, meats, vegetables and cereals (Ramachandran et al., 2006), whereas sodium gluconate is a derived salt produced through a neutralization reaction after the deep oxidation of glucose. These properties make them valuable in a variety of industrial applications; thus, they are gradually gaining public attention, including their use as acidity regulators and chelating agents in the food industry (Takano and Kondou, 2002; GÃ¼;nal, 2013), as moisturizing agents and pH regulators in cosmetics, as key components of concrete admixtures in the construction sector (Liu et al., 2024), as electrolyte supplements in pharmaceuticals, and as chelating agents for metal ions and essential components of detergents in chemical production. Furthermore, their environmentally friendly nature aligns with modern industry requirements for green chemicals, offering broad application potential.

In recent years, the quantity demanded of sodium gluconate has rapidly strengthened due to the wide application of sodium gluconate in the fields of food, medicine, construction, etc. (Opwis et al., 2008; Kundu et al., 2013; Tan et al., 2017), especially in the rapid expansion of emerging applications such as environmentally friendly detergents and concrete admixtures (Ma et al., 2015; Mota et al., 2019; Ma et al., 2024). It is reported that China’s sodium gluconate exports have exhibited strong growth momentum, with export volumes continuously increasing, positioning the country as a significant supplier in the global sodium gluconate market. According to customs statistics and industry analysis reports, China’s sodium gluconate exports have maintained an average annual growth rate exceeding 10% over the past 5 years, with products being exported to Europe, America, Southeast Asia, and various other countries and regions. From a global market perspective, the annual demand for sodium gluconate has surpassed 3 million tonnes, and this demand continues to expand at a compound annual growth rate of 5%–8%. In the face of the continuously growing market demand, the industrialized production of sodium gluconate is facing multiple challenges (Anonymous author, 2010). Besides the stable supply and quality control of the main raw material glucose, improving production efficiency and product quality through technological innovation in the production process is an urgent need to improve the production of sodium gluconate. At present, the traditional methods for producing sodium gluconate from glucose as a raw material include fermentation, catalytic oxidation, and biological enzyme methods, each of which has unique technical characteristics and application scopes. We compare several important indicators of these methods, as shown in Table 1. The fermentation method uses microorganisms such as *Aspergillus niger* to convert glucose into gluconic acid and then neutralize it to produce sodium gluconate (H.Blom et al., 2002; Prabu et al., 2012; Liu et al., 2017a). Although fermentation is a mature process and has relatively low investment in equipment, it has a long production cycle, high energy consumption, and limited product purity, and the byproducts produced during fermentation increase the difficulty of subsequent purification (Lu et al., 2016a). The catalytic oxidation method has rapidly developed in recent years through the direct oxidation of glucose to gluconic acid under the action of a catalyst and then neutralizes to obtain the target product. This method has the advantages of fast reaction speed, high production efficiency, and high product purity, but it requires strict requirements for the selection of catalysts and the control of reaction conditions, a large investment in equipment, and a high catalyst cost (Batcha et al., 2024). At present, precious metal catalysts are expensive despite their high activity (Liu et al., 2017b), while non-precious metal catalysts have a lower cost, but their activity and stability still need to be further improved. With the progress of biotechnology, the green production process is emerging as it uses dual enzyme method of glucose oxidase and catalase to generate gluconic acid. The advantages of this method include the absence of bacterial strains, independence from the concentration of various auxiliaries in the reaction solution, energy savings, optimization of reaction steps for ease of operation, high purity and easy separation of products, facilitation of purification, high product quality, short production cycles, and low pollutant emissions. Additionally, this method offers mild reaction conditions, good selectivity, and environmental friendliness, aligning with the development trend of green chemistry. Glucose oxidase (GOD) is an important oxidoreductase that can efficiently catalyze the oxidation of glucose to produce gluconic acid and hydrogen peroxide (H_2_O_2_) (Meng et al., 2014; Wang et al., 2022). Its catalytic mechanism comprises two consecutive steps: first, GOD oxidizes β-D-glucose to D-glucose-δ-lactone in the presence of molecular oxygen, using its cofactor FAD as an electron carrier, while FAD is reduced to FADH_2_; subsequently, D-glucose-δ-lactone is spontaneously hydrolyzed to gluconic acid through a non-enzymatic reaction (Hatzinikolaou et al., 1996; Bankar et al., 2009), while reduced FADH_2_ reacts with molecular oxygen to regenerate FAD, accompanied by the production of H_2_O_2_ (Leskovac et al., 2005). However, H_2_O_2_ causes oxidative damage to key residues such as methionine and cysteine in the active center of GOD, leading to enzyme inactivation, which significantly limits the catalytic efficiency of GOD. To address this issue, researchers developed a GOD/CAT multi-enzyme co-catalysis system. CAT efficiently and specifically catalyzes the decomposition of H_2_O_2_ into water and oxygen, with the generated oxygen being reusable in glucose oxidation reactions, thus establishing a virtuous cycle. In this system, the introduction of CAT not only effectively eliminates the inhibitory effect of H_2_O_2_ on GOD but also enhances the overall catalytic efficiency through a cascade reaction (Ruales-Salcedo et al., 2019; Cheng et al., 2023). Specifically, H_2_O_2_ produced by GOD was decomposed by CAT into water and 0.5 mol of oxygen, which could re-enter the catalytic cycle of GOD, significantly improving substrate utilization. Through this synergistic effect, the GOD/CAT system is able to operate stably at relatively high substrate concentrations, overcoming the production limitations of traditional single enzyme systems. In actual production, after glucose is catalytically oxidized by the GOD/CAT system to generate gluconic acid, the target product, sodium gluconate, can be obtained by adding NaOH for the neutralization reaction (Youcai et al., 2022). This synergistic multi-enzyme catalytic strategy not only improves the reaction efficiency but also reduces the risk of enzyme inactivation, which provides a reliable technological path for the green biomanufacturing of sodium gluconate.

**TABLE 1:** Sodium gluconate production methods.

Method	Reaction time	Product purity	Environmental friendliness	Applicable fields	Industrialization level
Biological Fermentation	36–60 h	>99%	Good	Food/Pharma/Construction	Mature
Homogeneous Chemical	2–4 h	∼95%	Poor	Industrial Grade	Being phased out
Electrolytic Oxidation	6–10 h	>98%	Good	Lab-scale	Niche
Heterogeneous Catalysis	6–8 h	>97%	Moderate	Industrial Grade	Partial application
Dual-Enzyme Conversion	<12 h	>99.5%	Optimal	Food/Pharma/Construction	Emerging technology

The bioenzymatic method represents the development direction of green production in the future, and with the progress of technology, its application prospects are broad. In the future, with the continuous optimization of the production process and breakthroughs in new technologies, the production of sodium gluconate will develop in the direction of greater efficiency, environmental protection, and economy. In addition, with increasingly stringent global environmental regulations and the promotion of the ‘double carbon’ goal, sodium gluconate manufacturers are facing pressure to save energy and reduce emissions. This situation has encouraged enterprises to increase investment in research and development to reduce energy consumption and emissions by enhancing production processes, optimizing equipment arrangements, and developing new catalysts. Additionally, growing demands for product purity and performance in downstream applications have driven manufacturers to consistently improve product quality and associated technologies. Looking forward, the sodium gluconate market is expected to maintain steady growth due to ongoing global economic expansion and the continuous emergence of new application areas (Ma et al., 2024). As a leading global manufacturer and exporter of sodium gluconate, China must further reinforce technological innovation, optimize industrial structures, and increase the added value of products to effectively address intensifying international competition and achieve a more advantageous position in the global supply chain. In the current study, sodium gluconate was produced using a dual enzyme approach in a 5 L fermenter, and the influences of parameters such as pH, temperature, metal ions, enzyme dosage, enzyme addition methods, stirring speed, aeration, and pressure were examined. Response surface methodology is applicable for determining optimal parameters in complex systems through mathematical modelling, regression analysis, and variance analysis (Gu et al., 2012; Tao et al., 2022). Based on single-factor test optimization, the Box-Behnken design (BBD) response surface experiment was performed to identify the optimal reaction conditions for sodium gluconate production by the dual enzyme technique and to further optimize the bio-enzymatic method (Liao et al., 2017).

## 2 Materials and methods

### 2.1 Experimental materials and instruments

The glucose oxidase (GOD) and catalase (CAT) used in the experiment were produced by Yiduoli (Guangdong) Biotechnology Co., Ltd.; glucose (food grade) was produced by Shandong Xiwang Group; sodium hydroxide (food grade) was produced by SINOPHARM Group Chemical Reagent Co., Ltd.; the reactor was a 5 L fermenter (Bailun Biologicals), equipped with temperature electrodes (Omega) and Mettler Dissolved oxygen electrode; pH electrode was produced by Shanghai Guoqiang Biochemical Equipment Co., Ltd.; UV-visible spectrophotometer (UV-2800A) was manufactured by Unico (Shanghai) Instrument Co., Ltd.; biosensor analyzer (SBA-40E) was manufactured by Jinan Yanhe Biotechnology Co.

### 2.2 Enzyme activity assay

#### 2.2.1 Glucose oxidase enzyme activity

Weigh 0.1 g of o-dianisidine into a small beaker and dissolve it in 10 mL of methanol. Then, take 1 mL of this mixture and dissolve it in 120 mL of phosphate buffer solution to prepare the o-dianisidine buffer solution (prepare immediately before use). A phosphate buffer solution with a pH of 5.6 was prepared, 2.54 g of dipotassium hydrogen phosphate and 12.45 g of potassium dihydrogen phosphate were accurately weighed, and a volume of 1 L was added. Subsequently, pipette 2.5 mL of the o-dianisidine buffer solution, 0.3 mL of 200 g/L glucose solution, and 0.1 mL of horseradish peroxidase solution into a clean test tube. Incubate the mixture in a 37°C water bath for 5 min. The mixture was removed, and 0.1 mL of o-anisidine buffer solution was added. In the test tube, 0.1 mL of the enzyme mixture to be tested was added, 0.1 mL of distilled water was added to the blank tube as a blank control, and the mixture was quickly shaken and poured into a cuvette at a wavelength of 460 nm. Absorbance changes were recorded at 1min intervals for 5min (Equations 1-3).

$$X\;\left(U/\text{mL}\right)=\left[\left(△A\times V2\right)/\left(11.3\times t\times V1\right)\right]\times n$$ (1)

$$△A_{n+1}=A_{n+1}–A_{n}\;\left(n=0,1,2,3,4,5\right)$$ (2)

$$△A=\left(△A1+△A^{2}+△A3+△A4+△A5\right)/5$$ (3)

V1: volume of enzyme solution to be tested, mL V2: volume of the reaction system, mL n: number of dilutions 11.3: reactant extinction coefficient t: enzymatic reaction time, min.

#### 2.2.2 Catalase enzyme activity

Catalase’ activity was detected using a biological test kit by Nanjing. The test tubes were numbered, and 0.1 mL of the sample along with 1 mL of Reagent I was added to each test tube. The tubes were then placed in a 37°C water bath for 3–5 min. During the test, 0.1 mL of Reagent II was added to each tube and mixed immediately. The mixture was precisely marked and immediately returned to the water bath to react for exactly 1 minute (60 s). After this, Reagent III was added to terminate the reaction, and the mixture was thoroughly mixed. The second and third tubes were processed sequentially, after which Reagent IV was added to all tubes and mixed well. The absorbance was measured using a spectrophotometer at a wavelength of 405 nm, with an optical path length of 0.5 cm (Equation 4). Double-distilled water was used as a reference (zero). The absorbance difference was calculated using equation: △A = A _control_ - A _measurement_.

$$\text{CAT viability }\left(U/\text{mL}\right)=\Delta A\times 271÷V_{\text{sample}}÷T\times N$$ (4)

271: reciprocal of slope, constant, used directly V _sample_: sample volume, 0.1 mL T: reaction time, 60 s N: sample dilution before testing.

### 2.3 Production of sodium gluconate

The reaction was carried out in a 5 L fermenter. First, 3 L of glucose solution (300 g/L) was added. After heating to the target temperature, aeration and stirring speed were adjusted to set the initial dissolved oxygen level to 100%. The pH was automatically controlled during the reaction by continuously adding a 30% NaOH solution. Specific amounts of GOD and CAT were then added, and samples were collected every 2 h. The glucose concentration was measured, and the OD values were recorded. The reaction ended once glucose concentration in all samples fell below 1 g/L, or when it decreased below 1 g/L within half an hour.

### 2.4 Detection of residual sugar

The quantity of residual sugar, i.e., the glucose concentration (g/L), was tested by an SBA-40C biosensor analyzer. First, a standard glucose solution of 100 mg/dL was used for the calibration operation of the instrument, and then, 25 μL of the diluted solution to be tested was sucked up and inserted into the inlet hole. Then, it was quickly punched in and pulled out, and readings were taken when the instrument displayed the readings (mg/dL). The glucose content was calculated on the basis of the dilution multiples (g/L).

### 2.5 Single-factor analyses

Based on the preliminary experiments, single-factor tests were done to study the effects of stirring speed, enzyme (GOD and CAT) addition, enzyme addition methods, and aeration on the reaction cycle. The stirring speeds tested were 500 rpm, 600 rpm, 700 rpm, and 800 rpm. Enzyme (GOD and CAT) amounts were 1.44 mL (0.16%), 1.62 mL (0.18%), 1.8 mL (0.2%), and 1.98 mL (0.22%). Four enzyme addition methods were compared: GOD added all at once; 80% GOD added at 0 h and 20% at 2 h; 80% at 0 h and 20% at 4 h; and 80% at 0 h and 20% at 6 h. Aeration rates tested were 0.5 vvm, 1 vvm, and 1.5 vvm (Jung et al., 2013; Velmurugan et al., 2022).

### 2.6 Box-Behnken design (BBD)

A BBD was used to optimize the reaction conditions based on the results from the single-factor tests. The reaction cycle was set as the response value, and three key factors (stirring speed, enzyme (GOD and CAT) addition, and aeration) affecting the sodium gluconate reaction cycle were selected as independent variables. A three-factor, three-level experiment was designed using Design-Expert 13 software, and the experimental factors and levels are shown in Table 1. Response surface methodology was applied to evaluate and optimize the reaction conditions, determining the best parameters (Nie et al., 2023).

### 2.7 Reaction cycle

The reaction was stopped when glucose concentration in all samples dropped below 1 g/L, or when it fell below 1 g/L within 30 min.2.8 Data processing.

All experiments were done three times in parallel. The results were averaged and plotted using Origin 2021. The response surface experiment was designed and analyzed using Design-Expert 13 software.

## 3 Results and discussion

### 3.1 Analysis of enzymatic properties

**FIGURE 1:**
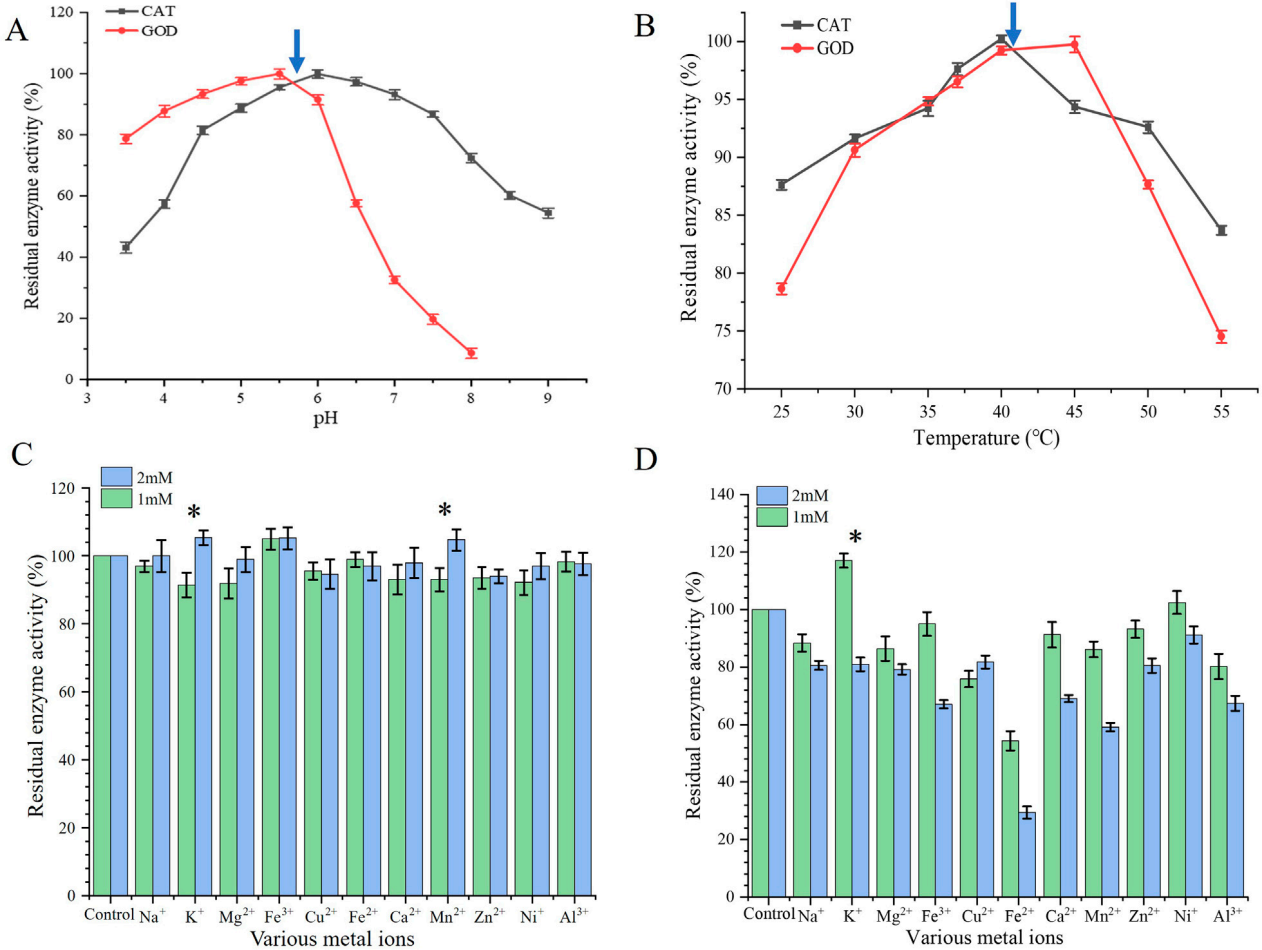
Effects on GOD and CAT enzyme activities. **(A)** Effects of pH; **(B)** Effects of temperature; **(C)** Effects of different metal ions on GOD activity; **(D)** Effects of different metal ions on CAT activity. Data represent the means ±SDs of duplicate reactions.

The enzyme activity is easily affected by pH. In order to investigate the optimum pH of GOD and CAT, we determined the enzyme activities of GOD and CAT at different pH values (3.5–9.0). As shown in Figure 1A, the enzyme activity of GOD increased with the increase in pH but decreased when the pH value was above 5.5; while for CAT, the enzyme activity reached a peak at pH 6.0. Besides, the temperature is another important factor for enzyme activity. We changed the temperature (25°C–55°C) to study the optimum temperature of the enzymes. As shown in Figure 1B, the optimum temperatures of GOD and CAT were 45°C and 40°C, respectively.

The effect of metal ions on the activity of GOD and CAT is shown in Figures 1C,D, where the control was the absence of metal ions (blank). For GOD, K^+^, Fe^3+^, and Mn^2+^ could promote enzyme activity, especially when the concentration was 2 mM, while most of the other metal ions did not promote enzyme activity. In addition, 1 mM K^+^ had a greater promoting effect on CAT, whereas Ni+ was also able to promote CAT; 2 mM metal ions did not promote CAT activity. The effects of different concentrations of metal ions on enzyme activity were somewhat concentration-dependent that showed more significant effects at higher concentrations for some metal ions. From the above conclusion, metal ions did not have a promoting effect to the glucose oxidase when the concentration at 1 mM and 2 mM; while for catalase, metal ions, especially at 1 mM, had a promoting effect on the catalytic activity.

### 3.2 Analysis of controlled conditions for the production of sodium gluconate by a dual enzyme system

**FIGURE 2:**
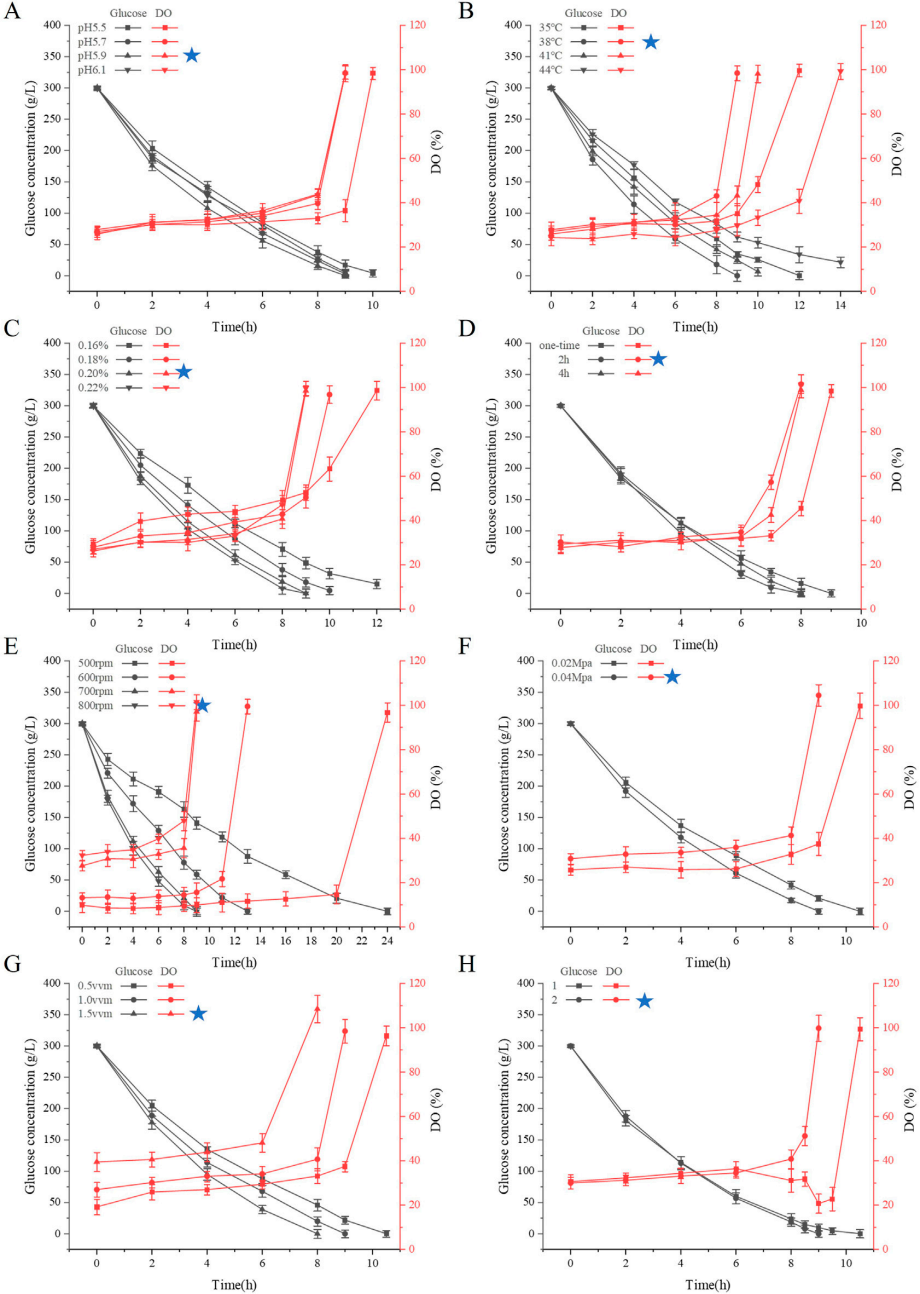
Effect of reaction conditions on the reaction cycle of the dual-enzyme GOD and CAT production of sodium gluconate. **(A)** Effects of pH; **(B)** Effects of temperature; **(C)** Effects of enzyme addition; **(D)** Effect of enzyme addition method; **(E)** Effects of agitation speed; **(F)** Effects of stress; **(G)** Effects of aeration rate; **(H)** Effects of frequency conversion on mixing speed. The data represent the means ±SDs of duplicate reactions.

The reaction conditions for the catalytic hydrolysis of glucose solution for the production of sodium gluconate by the GOD and CAT dual enzymes were investigated in a 5 L bioreactor. On the basis of the results obtained previously, the optimum pH range for the combination of GOD and CAT was between 5.5 and 6.0. Therefore, the effect of pH (5.5–6.1) on the number of reaction cycles was investigated (Figure 2A) with a stirring speed of 700 rpm, an aeration rate of 1 vvm, and a manometer value of 0.04. The reaction cycle at pH 5.9 was shorter than that at pH 5.5, 5.7, and 6.1, and the glucose content basically decreased to about 0 g/L at the end of the reaction. The initial dissolved oxygen concentrations all remained low, indicating that the reaction consumed a large amount of oxygen at this stage, and the DO concentration rapidly increased toward the end of the reaction, which could be used to determine whether the reaction was complete. Therefore, in the next reaction, the pH was fixed at 5.9, and regulated by sodium hydroxide solution.

The optimum reaction temperature for the combination of GOD and CAT was 35°C–44°C. In the present study, the reaction temperatures were controlled at 35°C, 38°C, 41°C and 44°C, and their effects on the reaction cycle and glucose consumption rate were investigated (Figure 2B). The reaction cycle with respect to the glucose consumption rate at 38°C was superior to that at 35°C, 41°C and 44°C. As the temperature increased, the amount of glucose residue increased, which might because the high temperature led to inactivation of the enzyme, and further led to the termination of the reaction, ultimately, the glucose was not completely converted into sodium gluconate. Therefore, controlling the reaction temperature at 38°C is favorable for increasing the reaction rate, ensuring high yield and protecting enzyme activity.

The effects of the amount of enzyme mixture (GOD or CAT) on the number of reaction cycles were investigated. The addition of GOD and CAT was maintained at a ratio of 1:1 at dosages of 1.44 mL (0.16%), 1.62 mL (0.18%), 1.8 mL (0.2%) and 1.98 mL (0.22%), and the temperatures were controlled at 38°C, pH 5.9, a stirring speed of 700 rpm, a venting ratio of 1 vvm, and a manometer value of 0.04. As the amount of enzyme added increased, the number of reaction cycles gradually decreased, and the glucose consumption rate gradually increased (Figure 2C). When 0.22% of the enzyme mixture was added, the reaction was completed within 8.5 h, and the final glucose concentration was less than 0.5 g/L. The reaction was carried out with 0.16% enzyme mixture. In contrast, the experiments were carried out with enzyme additions of 0.16% and 0.18%, and the results revealed that the reaction ended in the middle of the reaction, more glucose remained in the reaction mixture, and a decrease in the amount of enzyme had a greater effect on the number of conversion cycles. When the amount of enzyme added was 0.2%, the enzymatic reaction could still convert glucose into sodium gluconate more thoroughly, and the period of 9 h was slightly longer. In actual production, according to the actual process, comprehensive consideration of the cost is appropriate to reduce the amount of enzyme additive; thus, 0.2% enzyme additive was subsequently used for the experiment.

Considering the loss of enzyme activity during the reaction process, the relationship between the enzyme addition method and the number of reaction cycles was explored. The input methods were all one-time inputs (excess) for CAT, and the GOD was divided into one-time inputs and batch inputs. Three groups of experiments were designed, namely, one-time inputs for GOD; 0 h inputs for 80% GOD and 2 h inputs for 20% GOD; 0 h inputs for 80% GOD; and 4 h inputs for 20% GOD. The reaction cycle of the double enzyme was 9 h, and the residual sugar content was 0.05 g/L. The residual sugar content of the 2 h batch input enzyme was only 1.74 g/L at 7.5 h, and the reaction cycle of the 4 h batch input enzyme was 8 h (Figure 2D). It may be possible that the substrate concentration is high at the initial stage of the reaction, leading to high enzyme activity and content, while enzyme inactivation occurs gradually in the middle stage. When the total enzyme amount added is the same, batch enzyme addition results in a shorter reaction cycle and higher residual enzyme activity at the end. This residual activity allows further glucose conversion through replenishment, making better use of the enzymes. It can thus be concluded that batch addition of GOD effectively shortens the reaction cycle, but the timing and amount of enzyme addition must be carefully controlled.

The production of sodium gluconate is an aerobic reaction catalyzed by GOD, and therefore, effective oxygen supply promotes the reaction. The oxygen transfer rate in the system is closely related to agitation speed, tank pressure, aeration rate, physicochemical properties of the liquid, and reactor design. In this study, the influences of agitation speed, tank pressure, and aeration rate were investigated (Xiao-Ting et al., 2021; Sahlan et al., 2024).

To investigate the effect of stirring speed on the fermentation of sodium gluconate, four speed conditions were set in this experiment—500 rpm, 600 rpm, 700 rpm, and 800 rpm—and the rest of the reaction conditions were the same: 1.8 mL (0.2%) of each of the enzyme liquids, a temperature of 38°C, a pH of 5.9, an aeration ratio of 1 vvm, and a manometer value of 0.04. As shown in Figure 2E, at a stirring speed of 500 rpm, the reaction cycle occurred within 24 h; at a stirring speed of 600 rpm, the reaction cycle was shortened by 11 h, and the reaction basically occurred within 13 h. At a stirring speed of 700 rpm, the dissolved oxygen content reached 97.1% within 9 h. At this time, when the residual sugar content was <1 g/L, the reaction essentially ended. At a stirring speed of 800 rpm, the reaction cycle was shortened by an hour, and the reaction basically occurred within 8 h. The faster the stirring speed is, the greater the amount of dissolved oxygen in the system and the faster the corresponding reaction speed. With increasing stirring speed, the degree of oxygen transfer increases, the consumption rate of glucose increases accordingly, and the number of cycles is shortened (Tian et al., 2018). However, in practice, there is also the issue of energy consumption, as well as damage to the enzyme, which can be lost with strong agitation. Therefore, an agitation speed of 700 rpm is more appropriate.

To examine the connection between tank pressure and reaction cycle, a glass-bodied fermenter without internal pressure regulation was employed. The internal pressure of the fermenter was adjusted by changing the gauge reading of the pressure-reducing valve to either 0.02 or 0.04. The experimental outcomes are illustrated in Figure 2F. At a gauge setting of 0.02, the reaction cycle reached completion after 10.5 h, while at a gauge setting of 0.04, the reaction concluded earlier at 9 h. At the gauge value of 0.04, the dissolved oxygen measurement reached 104.5, exceeding the dissolved oxygen measurement of 99.8 recorded at the gauge value of 0.02. An appropriate increase in pressure in the fermenter effectively increased the amount of dissolved oxygen in the tank and shortened the reaction time.

Keeping the stirring speed, tank pressure, and other conditions consistent, with ventilation ratios of 0.5, 1, and 1.5 vvm, the reaction period under a ventilation ratio of 1.5vvm was 8 h. In the experiment with a ventilation ratio of 1vvm, the reaction period was 9 h. When the ventilation ratio was adjusted to 0.5vm, the reaction period was prolonged by another 1.5 h (Figure 2G). The above experimental results showed that the stirring speed had a more significant effect on the enzyme reaction cycle than pressure and aeration. When stirring speed decreased gradually, the influence of aeration became clearer. Aeration is another key factor that affects oxygen supply. Proper stirring speed, pressure, and increased aeration rates in actual production can enhance oxygen supply and allow for scaling up the current process.

This study aims to save energy and improve reaction efficiency. To achieve this goal, the stirring speed was adjusted at different stages of the reaction to lower energy consumption. Based on earlier experimental results, stirring speed was reduced at the later stages of the reaction because substrate concentration decreased, causing the reaction rate to slow down. Excess dissolved oxygen could not participate in the reaction, leading to wasted energy. Using the 700 rpm stirring speed in Figure 2E as a control, batch 1 reduced stirring speed to 600 rpm at 8 h, causing dissolved oxygen to decrease, and further reduced it to 500 rpm at 9 h, causing dissolved oxygen to decrease again. Batch 1 reached the reaction endpoint at 10.5 h, which was 1.5 h longer than the control. For batch 2, stirring speed was adjusted differently. The speed was reduced to 600 rpm at 8.5 h, and the reaction essentially finished at 9 h, which matched the reaction cycle of the 700 rpm control shown in Figure 2E,H. These results suggest that appropriately lowering stirring speed at the late stage of the reaction can reduce energy consumption without affecting the overall reaction time.

### 3.3 Single factor analysis

**FIGURE 3:**
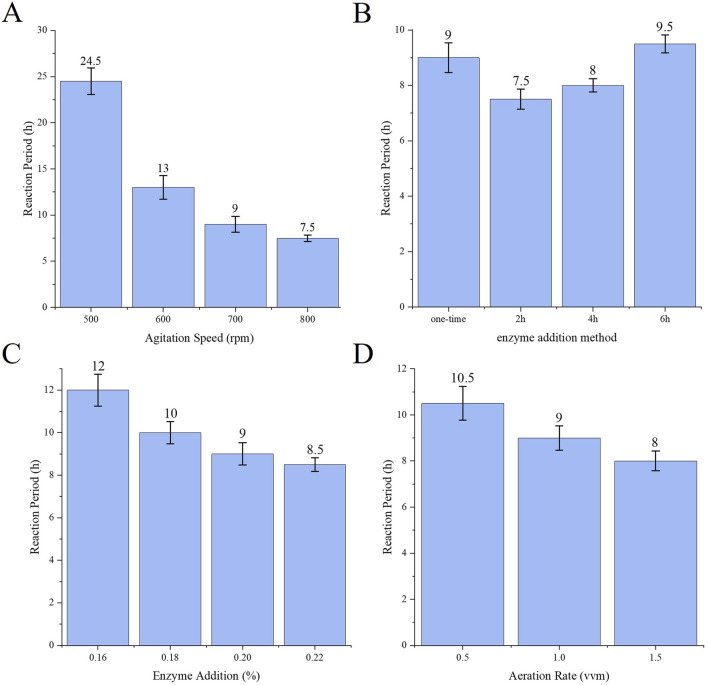
**(A)** Effect of stirring speed on reaction cycle **(B)** Effect of enzyme addition method on reaction cycle **(C)** Effect of enzyme addition amount on reaction cycle **(D)** Effect of aeration volume on reaction cycle.

The effect of each single factor on the reaction cycle for the production of sodium gluconate is shown in Figure 3. Figure 3A shows that with increasing stirring speed, the reaction cycle gradually becomes shorter because the change in stirring speed significantly affects the DO value during the reaction process, and with increasing stirring speed and increasing oxygen content, the rate of glucose consumption increases accordingly, and the number of cycles is shortened. As shown in Figure 3B, the enzyme addition method involves batch placement and replenishment at 2 h, and the reaction cycle is the shortest. This is due to the intense pre-reaction and excess enzyme amount, which partially inactivates the enzyme during the process. With respect to the total amount of enzyme added under the same conditions, the batch addition of the enzyme cycle is shorter. As the amount of enzyme decreased, more glucose remained in the reaction mixture, and as the number of cycles gradually increased, the amount of enzyme added had a greater effect on the number of conversion cycles (Figure 3C). Increasing the aeration volume can increase the rate of oxygen transfer to the reaction solution to shorten the number of reaction cycles (Figure 3D), but an aeration volume that is too high may lead to increased bubble incorporation or foam generation and affect the efficiency of oxygen transfer; thus, further selection of an appropriate aeration volume is needed.

### 3.4 Design of the tests by response surface

Based on the results of the single-factor experiments, a Box-Behnken Design (BBD) was employed, with stirring speed, enzyme addition, and aeration as independent variables. The response variable was the sodium gluconate reaction cycle. The experimental results, as presented in Table 3, were analyzed using multivariate regression analysis in Design Expert 13.0 software (Minneapolis, MN, United States).

$$Y=+12.71-7.94A-0.8375B-0.8438C-0.0125\text{AB}+0.0000\text{AC}-0.0625\text{BC}+4.50A^{2}-0.1925B^{2}-0.2050C^{2}$$

Seventeen experiments were performed using three independent variables, each at three levels (−1, 0, and +1), as shown in Table 2. Table 3 shows the results of these seventeen experiments, which were analyzed using Design Expert 13.0 software (Minneapolis, MN, United States). ANOVA was used to test the statistical significance of the quadratic regression equation (Table 4). The regression model had a p-value less than 0.0001, and the misfit term was not significant (p = 0.5348 > 0.05), showing that the regression equation obtained from the model was highly significant and fit the data well. The quality of the model fit was measured by the regression coefficient (*R* ^2^). The *R* ^2^ and adjusted *R* ^2^ values for the quadratic model were 0.9973 and 0.9939, respectively, which are very close to 1. The predicted *R* ^2^ (0.9809) agreed well with the adjusted *R* ^2^ (0.9939), with a difference less than 0.2. The Adeq Precision value, which measures signal-to-noise ratio, was 47.816. A value greater than 4 is considered good. This high ratio shows a strong signal, indicating that the actual and predicted values closely match. Thus, the model accurately describes the relationship between factors and response values in sodium gluconate production, and it can effectively predict optimal process conditions.

**TABLE 2:** Coded and actual values of factors in the Box-Behnken design.

Factor		Level	
	−1	0	1
(A) agitation speed	500	600	700
(B) enzyme addition	0.18	0.2	0.22
(C) aeration rate	0.5	1	1.5

**TABLE 3:** Response surface test design and results.

Test number	Agitation speed (rpm)	Enzyme addition (%)	Aeration rate (vvm)	Reaction period (h)
1	600	0.2	1	13
2	700	0.22	1	8.5
3	700	0.2	0.5	10
4	600	0.2	1	13.3
5	600	0.2	1	12
6	500	0.22	1	24.3
7	600	0.22	1.5	10.5
8	600	0.2	1	12.5
9	600	0.2	1	12.75
10	700	0.2	1.5	8
11	700	0.18	1	9.75
12	600	0.18	0.5	14
13	600	0.18	1.5	12.75
14	600	0.22	0.5	12
15	500	0.2	0.5	26
16	500	0.18	1	25.5
17	500	0.2	1.5	24

**TABLE 4:** Response surface regression model ANOVA.

Source	Sum of squares	Degrees of freedom	Mean square	F value	p Value	Significance
Model	601.24	9	66.80	290.88	<0.0001	***
A	504.83	1	504.83	2,198.14	<0.0001	***
B	5.61	1	5.61	24.43	0.0017	***
C	5.70	1	5.70	24.80	0.0016	***
AB	0.0006	1	0.0006	0.0027	0.9599	
AC	0.0000	1	0.0000	0.0000	1.0000	
BC	0.0156	1	0.0156	0.0680	0.8017	
A^2^	85.07	1	85.07	370.43	<0.0001	***
B^2^	0.1560	1	0.1560	0.6794	0.4370	
C^2^	0.1769	1	0.1769	0.7705	0.4092	
Residual	1.61	7	0.2297			
Lack of Fit	0.6256	3	0.2085	0.8495	0.5348	
Pure Error	0.9820	4	0.2455			
Cor Total	602.84	16				
R^2^ = 0.9973	R^2^adj = 0.9939	R^2^pre = 0.9809				
Adeq Precision	47.8164					

The values of “Prob. > F” values less than 0.001, 0.01, and 0.05 indicate that the model terms are extremely significant (***), respectively.

The degree of influence of each factor on the response cycle is different, with the F value indicating that the order of influence of the three factors is A (<0.0001) > C (0.0016) > B (0.0017), the interaction of AB, AC, and BC is not significant, and the items affected by the second factor, A^2^, have a very significant effect (P < 0.0001).

### 3.5 Analysis of response surface

**FIGURE 4:**
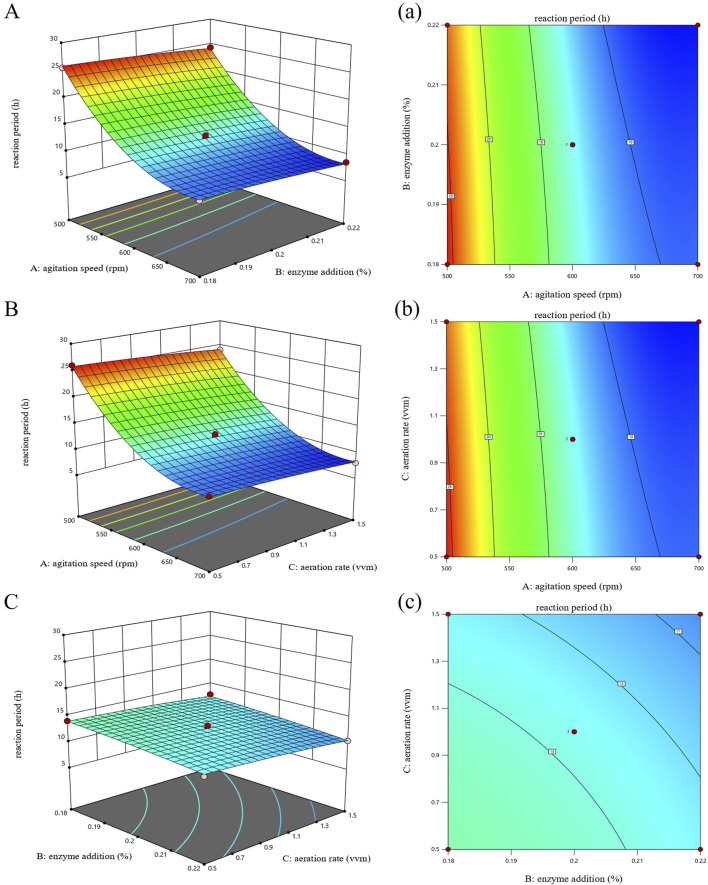
Response surface and contour plots for the sodium gluconate reaction period. **(A, a)** Effect of agitation speed and enzyme addition. **(B, b)** Effects of agitation speed and aeration rate. **(C, c)** Effects of enzyme addition and aeration rate.

To further analyze how stirring speed, enzyme addition, and aeration affect the reaction cycle, response surface and contour plots were created based on the regression equation, as shown in Figure 4. The contour plot is the horizontal projection of the response surface plot. These plots visually show the strength of interaction between factors: an oval-shaped contour indicates a strong interaction, while a round contour indicates a weak interaction. Additionally, the steepness of the response surface plot represents how much each factor influences the sodium gluconate reaction cycle; a steeper slope indicates a stronger effect. Based on the above plots and analysis, it can be concluded that interactions between factors were not significant. Among single factors, stirring speed had the greatest effect on the reaction cycle (P < 0.001), followed by enzyme addition and aeration (P < 0.01).

### 3.6 Optimization of response surface results

The experimental data were analyzed and optimized for prediction by Design Expert 13.0 software to obtain the optimum process with the shortest reaction cycle for the production of sodium gluconate at a stirring speed of 691 rpm, an enzyme addition of 0.21% and aeration of 1.2 vvm, and the predicted value of the reaction cycle for the production of sodium gluconate under these process conditions was 7.78 h. In the three sets of parallel experiments presented in Table 5, the reaction was validated in a 5 L fermenter under optimal conditions.

**TABLE 5:** Optimal fermentation conditions.

Reaction period (h)	Agitation speed (rpm)	Enzyme addition (%)	Aeration rate (vvm)
7.75	700	0.2	1.2

Based on the analysis of the regression model and practical conditions, a stirring speed of 700 rpm, enzyme addition of 0.2%, and aeration rate of 1.2 vvm were selected. Under these conditions, the measured reaction cycle for sodium gluconate was 7.75 ± 0.5 h, close to the predicted value from the model. This result confirmed that the model was reliable, and the regression equation could be effectively used for optimizing the sodium gluconate production process. There are several key challenges to scaling up this process to an industrial scale (e.g., 50 L or larger reactors). Firstly, the increased size of the reactor may lead to a decrease in the efficiency of oxygen transfer, requiring optimization of gas distribution or the use of segmented aeration to maintain dissolved oxygen concentration, while high agitation speeds (700 rpm) may lead to inactivation of the enzyme or excessive foaming due to excessive shear forces in larger equipment, requiring adjustment of the agitation method to balance efficiency and enzyme stability. Secondly, industrial-scale production requires higher costs and stability of the enzyme, and the reuse of the enzyme must be considered, for example, through immobilization techniques or optimized recycling processes to reduce costs. Meanwhile, metal ions in the feedstock may affect catalase activity, and it is necessary to purify the feedstock in advance or add protective agents (Han et al., 2018a). The cost required to produce one ton of sodium gluconate is simply calculated in Table 6, and it can be seen that the enzyme preparation accounts for about 2% of the cost. In addition, the complexity of process control increases significantly after industrial scale-up. Precise operations such as batch enzyme addition require precise control of addition timing, dosage and mixing effect, and dynamic adjustment of stirring speed requires maintaining high speed at the beginning of the reaction to ensure the efficiency of oxygen mass transfer, and then lowering the speed at the later stage to save energy and reduce enzyme shear damage, which puts forward higher requirements on the automation system, and it must rely on high-precision sensors to monitor key parameters such as glucose concentration, dissolved oxygen, pH and temperature in real time, and realize closed-loop automatic control through distributed control system. Glucose concentration, dissolved oxygen, pH and temperature and other key parameters, with fast response metering pumps, variable frequency motors and other actuators, and through the distributed control system to achieve closed-loop automatic control; At the same time, the reaction scale expansion leads to the exothermic heat release per unit of time dramatically increased while the tank wall heat dissipation efficiency is relatively low, it is very easy to cause high temperature, which leads to the enzyme irreversible inactivation, so it is necessary to strengthen the reactor heat transfer design, such as the use of large jackets, inner discs, and other equipment, to ensure the efficiency of oxygen mass transfer. Therefore, it is necessary to strengthen the heat exchange design of the reactor, such as the use of large jackets, internal coils or external plate heat exchangers, the use of low-temperature cooling water or refrigerant and other high-efficiency media, and optimize the hydrodynamics to ensure that the full mixing to eliminate the temperature dead space; to effectively solve these challenges of amplification, it is necessary to integrate the depth of the reactor sizing, mixing and heat exchange system design and other engineering optimization means, as well as based on the integration of multi-source data and process model prediction of advanced control algorithms, such as intelligent To effectively solve these amplification challenges, it is necessary to deeply integrate engineering optimization means such as reactor selection and heat exchange system design, as well as intelligent control strategies such as advanced control algorithms based on multi-source data integration and process model prediction, to realize dynamic optimization decision-making and precise implementation of key parameters such as mixing, aeration, enzyme addition and cooling through the synergy of hardware optimization and software intelligence, and finally guarantee the high-efficiency, stable and controllable operation of large-scale production to fully reflect the environmental protection and economic advantages of the process.

**TABLE 6:** Production cost calculation for 1 tonne of sodium gluconate.

Cost item	Consumption per unit	Unit price (RMB)	Unit cost (RMB)
Glucose	1.006804	85,120	3,157.55
Electricity	215	0.66	141.9
Steam	1.35	252	340.2
Glucose Oxidase	0.0016	19,500	31.6
Catalase	0.0016	19,500	31.6
Caustic Soda (Liquid)	0.6	760	456
Defoamer	0	11,000	0.23
Miscellaneous			230.3
Total Processing Cost			1,268.64
Total Production Cost			4,408.30

## 4 Conclusion

In this study, the reaction conditions were systematically optimized for the production of sodium gluconate from glucose via a dual enzyme method (GOD and CAT).

By analyzing the enzyme activities of GOD and CAT, the optimum pH was determined to be 5.5 and 6.0, and the optimum temperatures were 45°C and 40°C, respectively. A concentration-dependent study of metal ions revealed that the effects of some metal ions on enzyme activity were more significant at high concentrations, but the sensitivity of catalase to the concentration of metal ions should be noted. This study investigated batch enzyme addition and revealed that batch input of GOD could effectively shorten the reaction cycle and improve enzyme utilization, especially in the case of enzyme inactivation in the middle of the reaction, and batch enzyme addition could better maintain the reaction efficiency. In addition, an appropriate increase in tank pressure could improve the dissolved oxygen conditions and thus shorten the reaction time. Moreover, lowering the stirring speed at the later stage of the reaction could reduce energy consumption while ensuring that the reaction cycle is complete, which is valuable for application in practical production. Through one-way tests and response surface methods (Box-Behnken design), it was found that the stirring speed, enzyme addition, and aeration had a significant effect on the reaction cycle, among which the effect of the stirring speed was the most prominent, and a higher stirring speed accelerated the reaction rate; however, the inactivation of the enzyme due to excessive stirring should be avoided. In addition, the increase in enzyme addition could shorten the reaction cycle, and the increase in aeration could also help to improve the reaction efficiency. The optimal process parameters were as follows: pH, 5.9; temperature, 38°C; enzyme addition, 0.2%; the addition mode of CAT, one-time addition of GOD in batches; 80% GOD in 0 h; 20% GOD in 2 h; stirring speed, 700 rpm; aeration volume, 1.2 vvm; and pressure in the tank, 0.04 pa. The reaction time of sodium gluconate under these conditions was 7.75 ± 0.5 h, which significantly improved the efficiency of the reaction. By optimizing the process conditions for the production of sodium gluconate via the dual-enzyme method, this study significantly improved the reaction efficiency and reduced the production cost, which provides a theoretical basis and technical support for the industrial production of sodium gluconate. Compared with the traditional chemical method, the dual-enzyme method is more environmentally friendly, with milder reaction conditions and higher product purity. The dual enzyme system (GOD/CAT) realizes the green conversion of glucose to gluconic acid through efficient synergistic catalysis, and its core advantages are high specificity, self-detoxification ability and oxygen recycling. Despite the challenges of enzyme stability and cost, with the advancement of genetic engineering and material science, this system has great potential for application in food, medicine and biomanufacturing, and the future research direction will focus on the design of enzyme-material composite system and low-cost scale-up production process. And how to realize the biocompatibility of nanomaterials and reduce their potential toxicity remains a key challenge in research. Nanomaterial immobilized enzymes and nanoenzymes are significantly different in function and application; the former is an enzyme immobilized on the surface of nanomaterials to improve the stability and reusability of the enzyme, which is used in the fields of biosensors, industrial catalysis and environmental detection. The latter are nanomaterials with catalytic activity similar to that of natural enzymes and do not contain biological enzyme components, which are mostly used in biomedical diagnostics, environmental remediation and food safety testing. They can be used in harsh environments due to their high chemical stability, ease of production, applicability to a wider range of pH and temperature, and the shortcoming is that the catalytic efficiency and specificity are usually lower than that of natural enzymes. The innovative ‘batch enzyme addition’ strategy and optimized oxygen supply (e.g., adjusting tank pressure and aeration) in the study effectively reduced the reaction time from 10–12 h to about 7.75 h while saving energy consumption. However, challenges may be encountered when scaling up to industrial scale (e.g., 50-L or larger reactors), such as insufficient oxygen supply, decreased stirring efficiency, or poor enzyme stability. More efficient oxygen supply methods (e.g., air-lift reactors) and optimized stirring designs to reduce enzyme damage can be used to address these challenges. It also further explores enzyme immobilization technology (Ozyilmaz and Tukel, 2007; Zhao et al., 2018; Zhuang et al., 2019), reactor design optimization, and enzyme stability enhancement during the reaction process to achieve a more efficient and energy-saving production mode while reducing environmental pollution. In the future, if it can be combined with automated control and continuous production, this process will be more competitive and promote the wide application of sodium gluconate in various fields (Lu et al., 2016b).

## Data Availability

The original contributions presented in the study are included in the article/supplementary material, further inquiries can be directed to the corresponding author.
